# Identification of Key Candidate Genes and Chemical Perturbagens in Diabetic Kidney Disease Using Integrated Bioinformatics Analysis

**DOI:** 10.3389/fendo.2021.721202

**Published:** 2021-09-07

**Authors:** Zhuo Gao, Aishwarya S, Xiao-mei Li, Xin-lun Li, Li-na Sui

**Affiliations:** ^1^Department of Nephrology, Air Force Medical Center, Beijing, China; ^2^Department of Bioinformatics, Stella Maris College (Autonomous), Chennai, India

**Keywords:** diabetic nephropathy, gene ontology, prognosis, biomarkers, differential gene expressions, diabetic kidney disease

## Abstract

Globally, nearly 40 percent of all diabetic patients develop serious diabetic kidney disease (DKD). The identification of the potential early-stage biomarkers and elucidation of their underlying molecular mechanisms in DKD are required. In this study, we performed integrated bioinformatics analysis on the expression profiles GSE111154, GSE30528 and GSE30529 associated with early diabetic nephropathy (EDN), glomerular DKD (GDKD) and tubular DKD (TDKD), respectively. A total of 1,241, 318 and 280 differentially expressed genes (DEGs) were identified for GSE30258, GSE30529, and GSE111154 respectively. Subsequently, 280 upregulated and 27 downregulated DEGs shared between the three GSE datasets were identified. Further analysis of the gene expression levels conducted on the hub genes revealed SPARC (Secreted Protein Acidic And Cysteine Rich), POSTN (periostin), LUM (Lumican), KNG1 (Kininogen 1), FN1 (Fibronectin 1), VCAN (Versican) and PTPRO (Protein Tyrosine Phosphatase Receptor Type O) having potential roles in DKD progression. FN1, LUM and VCAN were identified as upregulated genes for GDKD whereas the downregulation of PTPRO was associated with all three diseases. Both POSTN and SPARC were identified as the overexpressed putative biomarkers whereas KNG1 was found as downregulated in TDKD. Additionally, we also identified two drugs, namely pidorubicine, a topoisomerase inhibitor (LINCS ID- BRD-K04548931) and Polo-like kinase inhibitor (LINCS ID- BRD-K41652870) having the validated role in reversing the differential gene expression patterns observed in the three GSE datasets used. Collectively, this study aids in the understanding of the molecular drivers, critical genes and pathways that underlie DKD initiation and progression.

## Introduction

Diabetes is a major global health crisis projected to affect 642 million people by 2040 (1). According to the statistics from the World Health Organization (WHO), the rapid rise in the population prevalence in low- and middle- income countries led to the dramatic rise in the number of diabetic patients from 108 million in 1980 to 422 million in 2014. Unfortunately, nearly 40 percent of all diabetic patients develop serious diabetic kidney disease (DKD) (2). The dangerous liaison between diabetes and DKD is well-established. Currently, diabetes is one of the leading causes of both end-stage renal disease (ESRD) and chronic kidney disease (CKD) (3, 4). According to one study, the mortality due to the DKD rose by 94 percent between 1990 and 2012 (5).

In 2015, the Global Burden of Disease (GBD) assessed 1.2 million deaths and 19 million disability-adjusted life-years attributed to the hostile ailments of diabetes directly related to the reduced glomerular filtration rates (6, 7). According to the data from the WHO, nearly 100 million Chinese people are currently suffering from diabetes (8). Several studies reported the prevalence and risk of CKD and DKD in Chinese residents. Liu et al. conducted a cross-sectional survey including 23,869 clinical samples to measure the prevalences and risk factors associated with CKD and DKD in a Chinese rural population (9). In participants with diabetes, the overall prevalence of CKD was more than 35 percent. The Chinese cohort study conducted from 2003 through 2015 implicated a higher mortality rate and shorter life expectancy in the patients suffering from DKD. Taken together, there is an urgent need to develop the methods for early detecting the progression of kidney diseases in diabetic patients.

Based on anatomical distinction between the different sections of human kidney, the DKD which is also commonly known as diabetic nephropathy (DN) are subdivided into glomerular or tubular (10). Henceforth, the diseases associated with the glomerular and tubular region will be called glomerular diabetic kidney diseases (GDKDs) and tubular kidney diseases (TDKDs). The GDKDs are characterized by reduced glomerular filtration capacity, and the deposition of immune leading into the damage of the glomerular basement membrane (11) whereas the TDKDs are characterized by the dysfunctions of transporters and channels in the renal tubular system, and the destructions of the normal tubular tissues (12).

The diagnosis of the DKD has posed a challenge due to the complicated etiopathology and comorbidity associated with other related diseases. Identification of both prognostic and diagnostics biomarkers to monitor the initiation and progression of DKD has been recommended (13). Several diagnostic biomarkers mainly identified by monitoring the morphological changes in diabetic kidney were reported. The marker of tubular damage such as neutrophil gelatinase-associated lipocalin (NGAL), alpha-1-microglobulin, kidney injury molecule-1 (KIM-1), N-acetyl-beta-D-glucosaminidase, angiotensinogen, uromodulin, liver-type fatty acid-binding protein (L-FABP), and transferrin were also reported (14). However, the prognosis of the disease is still poorly understood. To this end, gene expression profiling can aid tremendous value in identifying the putative prognosis biomarkers.

The integrated use of differential gene expression (DEG) analysis and bioinformatics approaches have been advocated in early detection and monitoring of disease progression. It has also been used in identifying the chemical perturbagens capable of intervening the critical molecular pathways in diseased conditions. For example, the genomics and transcriptomics data profiling has helped in elucidating the pathogenesis of diabetes (15–17) and in identifying potential new mechanisms in DN (18, 19). However, there are only a few studies that shed light on the potential pathogenesis mechanism and cross-talks between GDKD, TDKD and early diabetic nephropathy (EDN). For example, Woroniecka et al. conducted the transcriptomics profiling of GDKD and TDKD on human kidney samples (20). The integration of multiple genomics data and their reanalysis offers a great avenue to identify certain disease-related biomarkers and help overcome the pitfalls of single cohort study, poor reproducibility and consistency (21).

In the current study, we analyzed the gene expression profiles of EDN (GSE111154) (22), GDKD (GSE30528) (20) and TDKD (GSE30529) (20) accessed from the NCBI‐Gene Expression Omnibus database (NCBI‐GEO). We identified the differentially expressed genes (DEGs) and further examined the molecular factors that drive the DKD pathogenesis using an integrated bioinformatics approach. We further conducted the drug perturbation analysis using the LINCS L1000 data repository (23) to study the effect of small-molecules and identified the potential drug candidates which have the ability to induce and reverse the transcription profile signatures.

## Materials and Methods

### Dataset and Identification of DEGs

The high throughput gene expression profile datasets of EDN (GSE111154) (22), GDKD (GSE30528) and TDKD (GSE30529) (20) were obtained from NCBI-GEO (24). The datasets GSE30528 and GSE30529 were the subseries of GSE30122 and based on the GPL571 platform- HG-U133A_2 Human Genome Affymetrix array. GSE111154 was based on the platform GPL17586- [HTA-2_0] Affymetrix Human Transcriptome Array 2.0. The GDKD dataset had 9 samples and 13 samples collected from the glomerular tissues of DKD and normal patients, respectively. The TDKD dataset had 10 samples and 12 samples collected from tubular tissues of DKD and normal patients, respectively. The EDN dataset consisted of 4 clinical samples of the early DN and 4 non-diabetic control samples obtained from the kidney tissues and blood. The GEO2R module of the bioconductor package was used to identify DEG. The datasets were standardized, normalized by TPM (Transcripts Per Kilobase Million) and log transformed to identify DEGs with respect to the diseased *vs* control samples. In this study, the genes with log2FC > 1.5 and P value < 0.05 were defined as DEGs (25, 26). The datasets were examined both individually and in groups to identify the genes unique to the respective diseases. Volcano plots and heatmap clusters of the DEGs were plotted using the ggplot modules of R package 3.5.8 and R studio 1.4.1106 (27).

### Gene Ontology and Pathway Enrichment Analysis

The functions of the candidate DEGs were annotated using the DAVID gene annotation tool (https://david-d.ncifcrf.gov/) (28). The annotations were evaluated for three sub-ontologies *viz.* biological process (BP), molecular functions (MF) and cellular components (CC). The pathways enrichment analysis was performed using the Kyoto Encyclopedia of Genes and Genomes (KEGG) (29, 30) and REACTOME (31) databases. The interrelation analysis of the molecular pathways were conducted using the ClueGo (version 2.5.7) (32) implemented in Cytoscape software (version 3.8.2) (33). In this work, we have plotted the clusters based on the Benjamini Hochberg statistical significance including enrichment/depletion hypergeometric tests and a kappa score cutoff of 3 (21).

### Protein-Protein Interaction (PPI) Network Establishment and Modular Analysis

The DEG-encoded proteins and their interactions amongst each other were established using the STRING database (http://stringdb.org/) (34). The PPI-network was created and evaluated using the Cytoscape software. Additionally, the significant interactions within the PPI-network were screened using MCODE plugin (35) implemented in Cytoscape software. The selections were made based on the highest MCODE score, degrees and the number of nodes. In this work, the significant clusters were selected based on the network scoring degree cutoff of 2. The parameters to find the clusters were optimized to the haircut option with K- score cutoff of 2 and node score cutoff of 0.2.

### Validation of Hub Genes

The expression levels of the hub genes in various tissues were investigated using the expression atlas platform (https://www.ebi.ac.uk/gxa/home) (36). The presence and absence of the genes were considered as the biomarker for the kidney diseases based on the standard expression levels of the genes in the renal and associated systems. The protein expression levels of the hub genes were also identified from the protein atlas database (www.proteinatlas.org). The tissue pathology, transcript expression of hub genes as identified from Genotype tissue Expression dataset (GTEx) and their cell specific protein expression from human proteome atlas (HPA) in normal cells of glomeruli, and tubules were predicted.

### Confirmation of Hub Genes

The hub genes identified needed confirmation and hence a single-cell transcriptome data of early diabetic nephropathy (37) from Humphreys Lab (http://humphreyslab.com/SingleCell/) was analyzed with the data visualization tool. The dataset with GEO accession GSE131882 had an integrated single-nuclear (sn) RNA data that included 3 control and 3 early diabetes samples from podocytes (PODO), proximal convoluted tubules (PCT), cells of the loop of Henle (LOH), connecting tubule cells (CNT), distal convoluted duct cells (DCT), principal cells of collecting duct (CTP), glomerular endothelial cells (EDC), intercalated cell B from collecting duct (CTB), intercalated cell A from the collecting duct (CTA), parietal epithelial cells (PEC), messenger cells and immune cells (38). The hub genes were compared to the snRNA dataset to confirm their potential effect. Their expression in diabetic nephropathy *vs* healthy human donor was predicted through Nephroseq v5 tool (https://nephroseq.org). The cut off thresholds were p value of 0.05, and log2FC of 1.5 to filter out the expression patterns of the putative candidate biomarker genes.

### Drug Perturbation Analysis

We have conducted the transcriptomic signature similarity search on the identified DEGs using L1000FWD web server (http://www.lincsproject.org/LINCS/dmoa). The potential drug candidates which mimicked and reversed the gene expression signatures were ranked based on the similarity score close to 1 and -1 (39).

## Results

### Identification of DEGs in DKD

**Figure 1:**
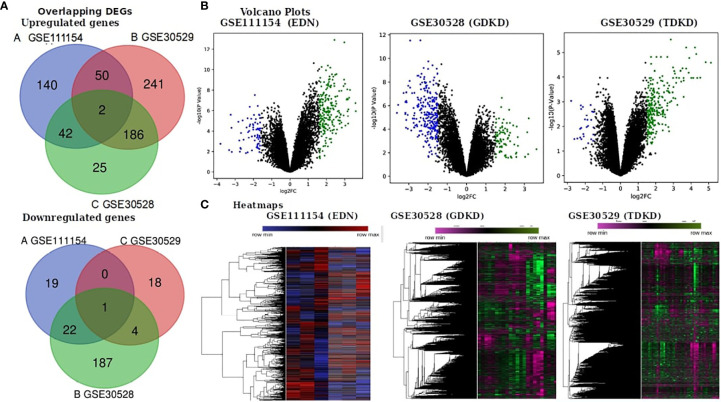
Identification of differentially expressed genes (DEGs) in the three datasets (EDN: GSE111154, GDKD: GSE30528 and TDKD: GSE30529). **(A)** Venn diagrams representing commonly changed DEGs in the three datasets. **(B)** Respective volcano plot of the three datasets. Red and blue plots represent up- and downregulated genes, respectively (log2FC > 1.5 and P value < 0.05). Black plots represent the remaining genes with no significant difference. **(C)** Heatmap of the top 500 DEGs are shown.

We obtained the gene expression profiles of diseased and controlled samples of GDKD, TDKD and EDN from the GSE30528, GSE30529 and GSE111154 datasets and identified the DEGs using the GEO2R package. Upon setting the cut-off criterion as log2FC > 1.5 and P < 0.05, we identified 1,241, 318 and 280 DEGs from GSE30528, GSE30529 and GSE111154, respectively. The overlapping DEGs between the three datasets were also identified (**Figure 1A**) The volcano plots of the DEGs of each dataset are shown in **Figure 1B**. The heat map of the DEGs based on Hierarchical clustering between patients with DKD and healthy controls are shown in **Figure 1C**. We have identified 44 upregulated and 23 downregulated genes overlapped between the EDN and GDKD, 188 upregulated and 5 downregulated genes were found overlapped between the GDKD and TDKD, and 52 upregulated genes were found commonly between the EDN and TDKD. Interestingly, two upregulated genes SULF1 and DCN, and one downregulated gene PTPRO were observed as common to the three datasets (**Figure 1A**). The overall statistics are shown in **Table 1**.

**Table 1:** Identification of the commonly changed DEGs in the three datasets.

Group	EDN *vs* GDKD	GDKD *vs* TDKD	EDN *vs* TDKD	EDN *vs* GDKD *vs* TDKD
Upregulated genes	186	42	50	2
Downregulated genes	22	4	0	1

### GO Term Enrichment Analysis of DEGs

Gene ontology enrichment is significant to elucidate the mechanisms of differentially expressed genes and we performed DEG GO analysis using the DAVID (https://david-d.ncifcrf.gov/) gene annotation tool. Three sub-ontologies *viz.* BP, MF and CC were examined. The DEGs of the respective datasets were examined and based on the *P-* value, the top 10 best ranked gene annotations were considered for further analysis (40). The GO annotations for the up- and downregulated genes are shown in **Supplementary Figures 1** and **2**, respectively. As shown in **Supplementary Figure 1** illustrating the BP term, the upregulated genes were mainly enriched in extracellular matrix organization, regulation of cell migration, response to cytokine stimulation, regulation of coagulation and glomerular epithelial cell differentiation; downregulated genes were largely enriched in regulation of glomerular filtration, chemotaxis and cellular response to vascular endothelial growth factors. For MF, enrichment of upregulated genes were mainly involved in the metalloendopeptidase inhibitor activity, tau protein binding, integrin binding and prostaglandin E receptor activity, and that of downregulated genes were mainly in the filamin binding, VEGF (Vascular Endothelial Growth Factor) receptor binding and CMP alpha 2-3 sialyltransferase and immunoglobulin binding mechanisms. For CC, enrichment of upregulated genes were primarily in the phagocytic vesicles and granular lumen and the downregulated genes mainly in secretory vesicles, exocytic vesicles, platelet alpha granules and endolysosomes. We have also performed the GO term enrichment analysis for the overlapped genes (**Supplementary Table 1**).

### Signaling Pathway Enrichment Analysis

**Figure 2:**
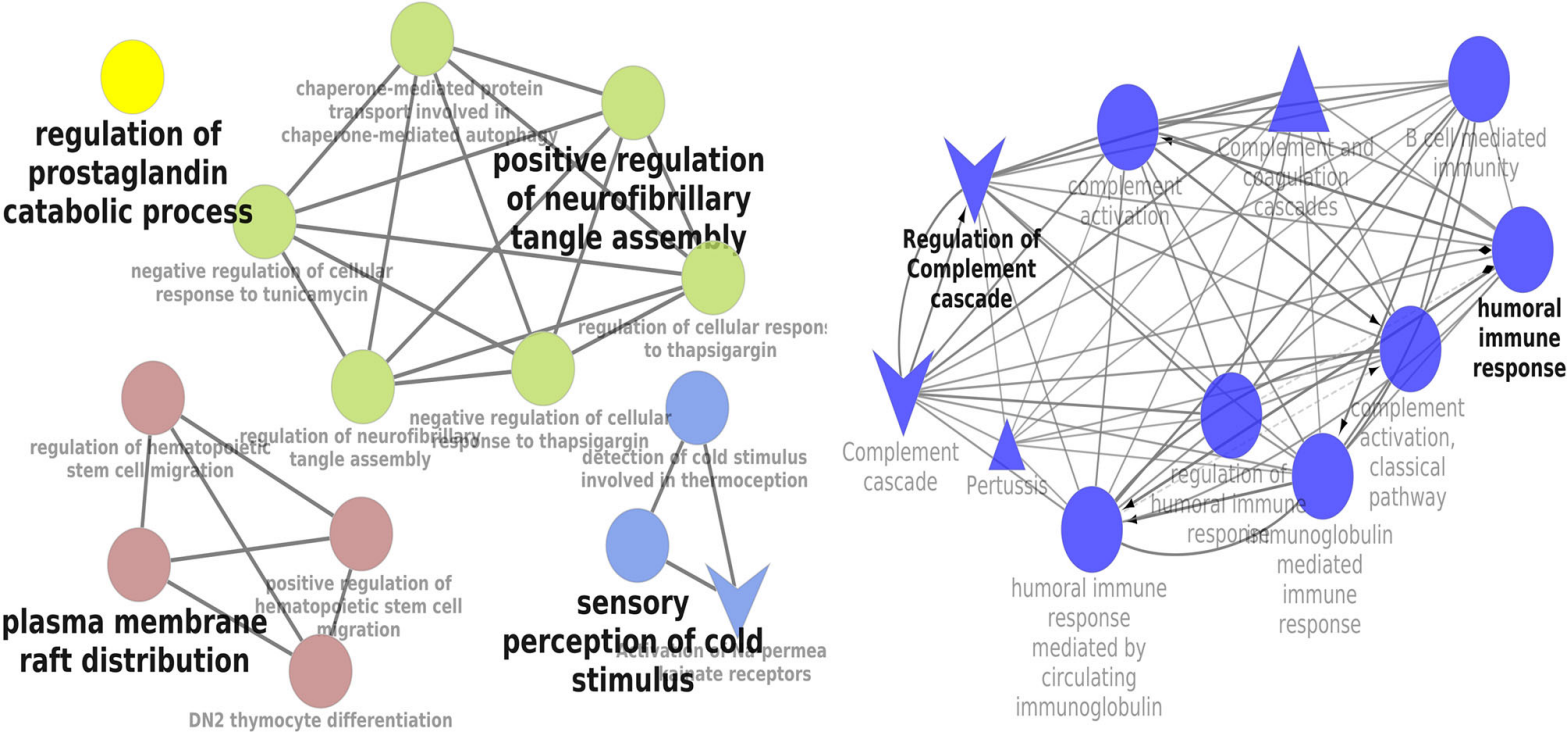
Interrelation analysis of pathways upregulated DEGs by examining KEGG processes in ClueGO.

**Figure 3:**
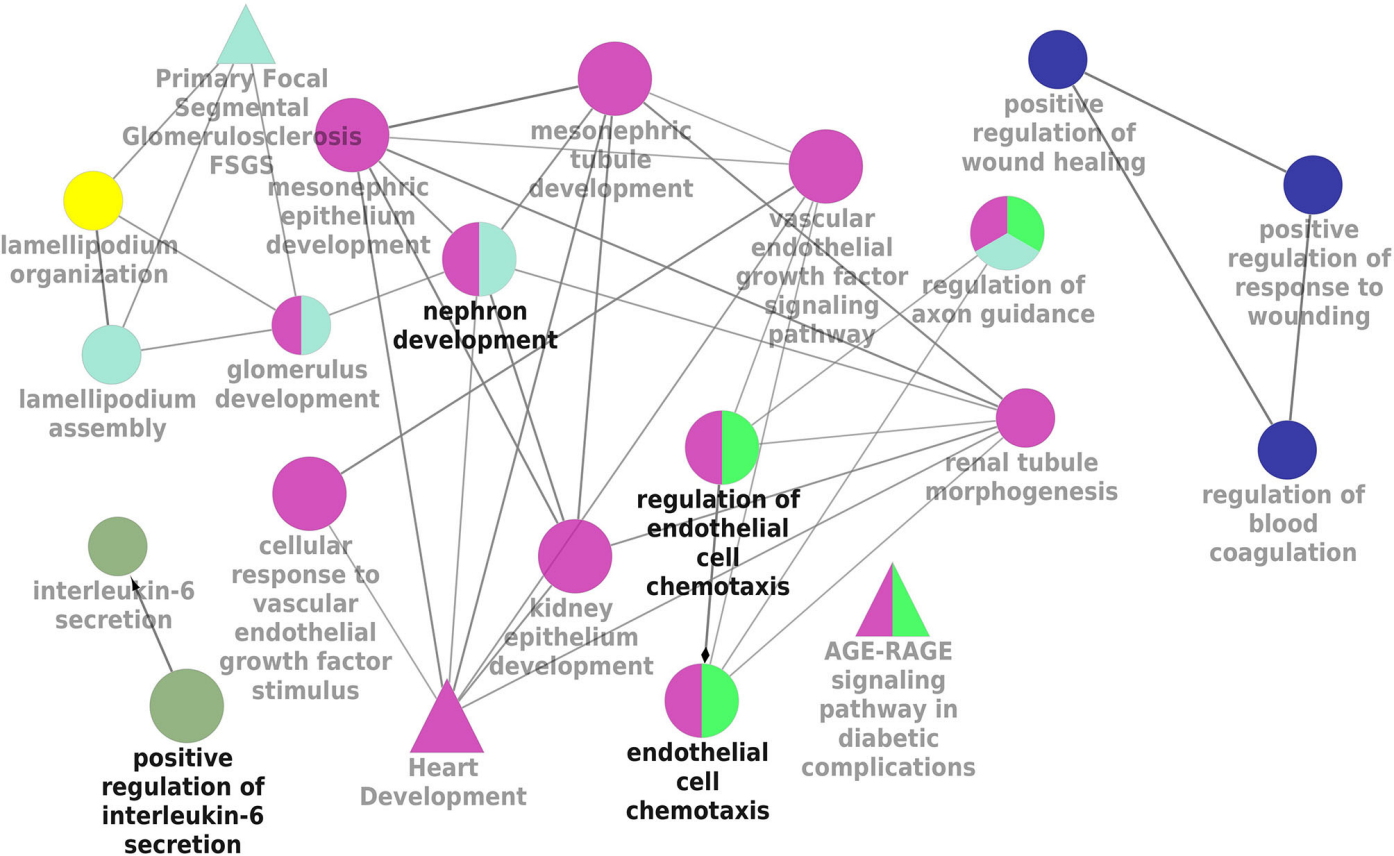
Interrelation analysis of pathways downregulated DEGs by examining KEGG processes in ClueGO.

KEGG and REACTOME pathway enrichment analyses of the DEGs (both up- and downregulated) were performed (**Supplementary Figure 3**). The significantly upregulated pathways were identified to be the hemostasis, aggregation and activation of platelets, collagen formation and complement cascade activation. Enrichment of downregulated DEGS was mostly in vascular endothelial growth factor receptor dimerization, VEGF- ligand interactions, defective B3GALTL (Beta 3-Glucosyltransferase) and pathways associated with O-glycosylation. We further performed the interrelational analysis of pathways by examining KEGG processes of up- and downregulated DEGs in ClueGO. Interestingly, the upregulated DEGs were primarily associated with the complement activation, collagen formation, collagen biosynthesis, humoral immune response, regulation of prostaglandin, perception of neurofibrillary tangle, cold stimulus and nephron morphogenesis (**Figure 2**). The downregulated DEGs were primarily associated with the regulation of axon guidance, endothelial cell chemotaxis, vascular endothelial growth factor signaling, MAP kinase signaling, protein kinase activity, glomerular filtration, collagen biosynthesis, formation of fibrin clots and coagulation (**Figure 3**).

### PPI Network Construction and Identification of Hub Genes

**Figure 4:**
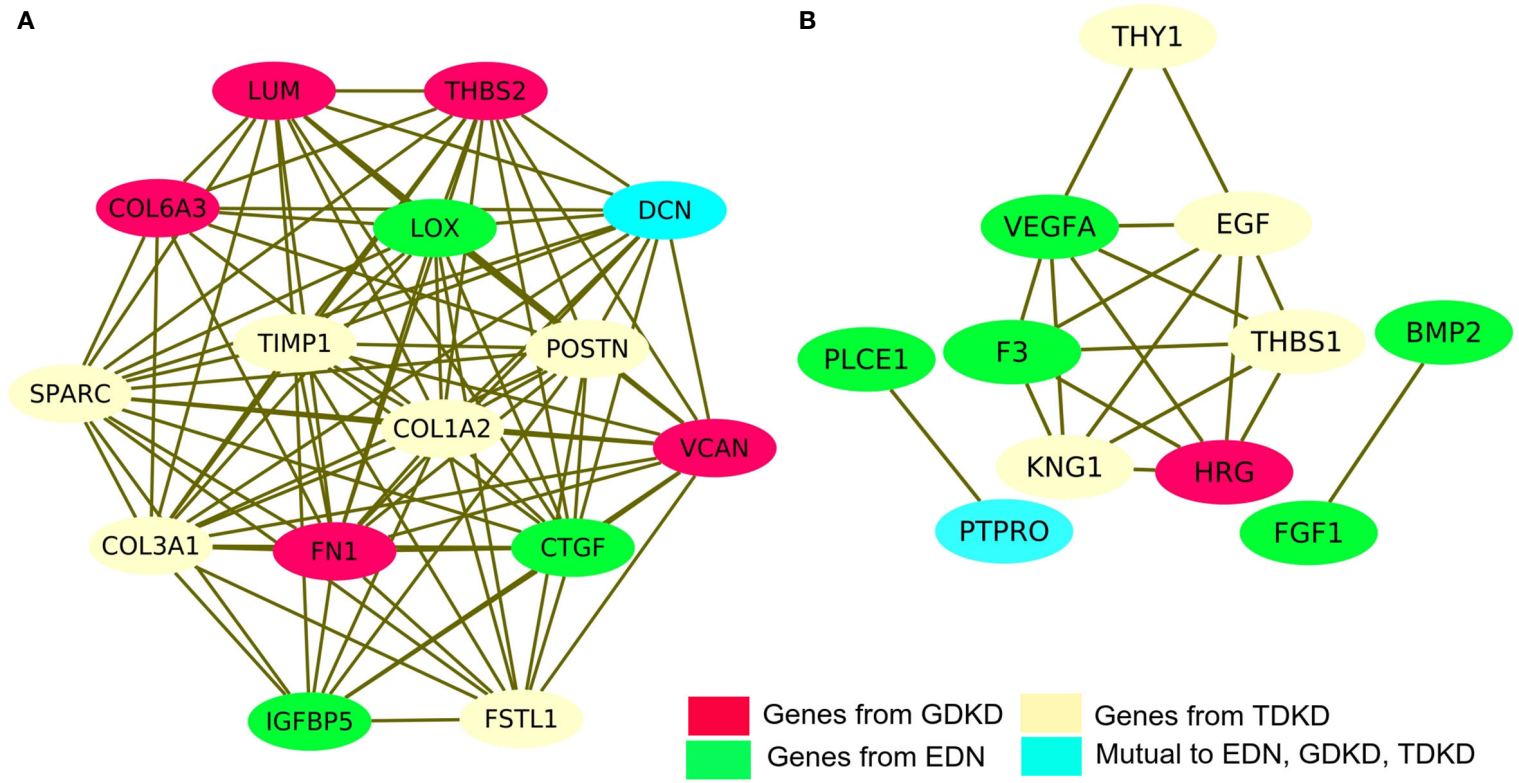
Protein–protein interaction (PPI) network of DEGs and module analysis. **(A)** Identification of a significant module based on the degree of importance examined for upregulated DEGs. **(B)** Identification of a significant module based on the degree of importance examined for downregulated DEGs. The representations are as follows: the potential genes unique to EDN, GDKD, and TDKD are shown in green, pink and cream colors respectively. The cyan color represents the common gene noticed in all three datasets for the respective up- and downregulated DEGs.

We have investigated the integrated pathways and PPI of the DEGs using the STRING database and Cytoscape software. The overall interactions were further validated with the MCODE module (shown in **Figure 4**). Significant PPI clusters for up- and downregulated DEGs were identified with the maximum score of 13.286 and 6.25, respectively. The PPI cluster of the upregulated DEGs included 15 nodes with 94 degrees. The PPI cluster of the downregulated DEGs included 7 nodes with 25 degrees along with two subclusters containing 2 nodes and 2 degrees each with a score of 3.32. CTGF (Cellular Communication Network Factor 2), IGFBP5 (Insulin Like Growth Factor Binding Protein 5) and LOX (Lysyl Oxidase) were identified as two upregulated hub genes for EDN. THBS2 (Thrombospondin 2), LUM, COL6A3 (Collagen Type VI Alpha 3 Chain), FN1 and VCAN were identified as four upregulated hub genes for GDKD. FSTL1 (Follistatin Like 1), TIMP1 (TIMP Metallopeptidase Inhibitor 1), POSTN, SPARC and COL3A1 (Collagen Type III Alpha 1 Chain) were identified as five upregulated hub genes for TDKD. VEGFA, PLCE1 (Phospholipase C Epsilon 1), BMP2 (Bone Morphogenetic Protein 2) and FGF1 (Fibroblast Growth Factor 1) were identified as four downregulated hub genes for EDN. HRG (Histidine Rich Glycoprotein) was identified as the downregulated hub gene for GDKD. Interestingly, two most significant candidate genes viz. DCN (encodes decorin) and PTPRO (encodes receptor-type tyrosine-protein phosphatase O) were found common for the respective up- and downregulated DEGs in all three datasets. Non overlapping upregulated gene COL1A2 (Collagen Type VI Alpha 2 Chain) and downregulated THY1 (Thy-1 Cell Surface Antigen), KNG1, EGF and THBS1 were also identified from the string interactions.

### Validation of Hub Genes

**Figure 5:**
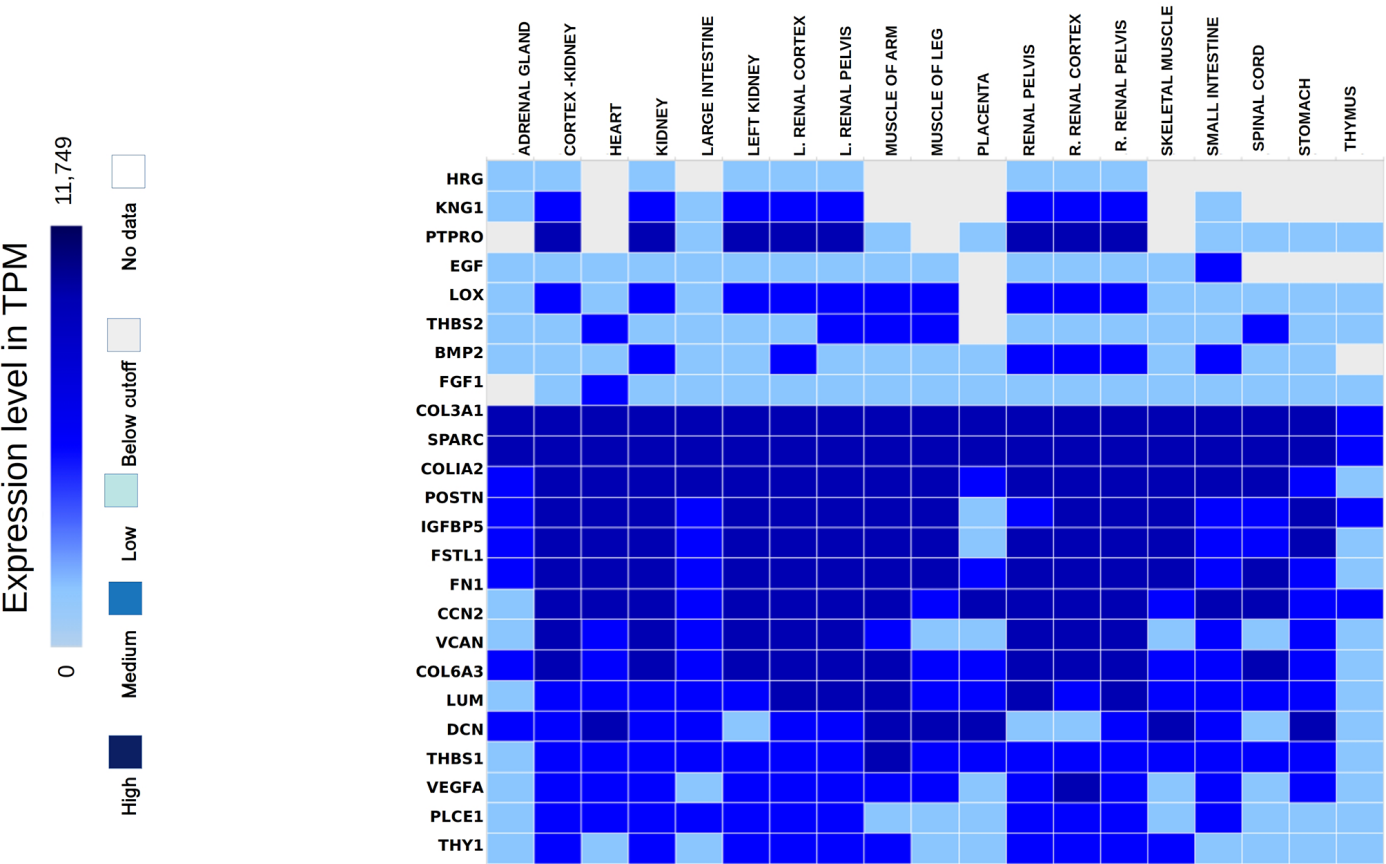
Hub genes identified from the expression atlas based on their transcripts per kilobase million expression level in the renal system.

The hub genes were validated based on the comparison between the standard expression levels from the Genotype tissue expression (GTEx) dataset and the fold change as a measure of Transcripts per kilobase million (TPM) with a maximum of 11,749 TPM. Analysis of the distribution of the hub genes revealed that they were significantly expressed in both left and right cortex tissues of the kidney. As per the GTEx dataset, higher expression levels of PTPRO, NPHS1, VCAN, COL6A3, THBS1 and FN1 were found in the cortex and pelvis of both the kidneys. Medium expressions of KNG1, LOX, THY1, THBS2, PLCE1 and VEGFA and lower expressions of HRG were identified in the renal tissues (**Figure 5**). Validation was further extended with the prediction of the tissue specific expression rates as per GTEx data set and cell specific expression rates from Human Protein Atlas maps and are represented in **Table 2**.

**Table 2:** Expression levels of the hub genes identified from GTEx and HPA data.

Hub genes	RNA seq expression	Protein Expression		Sn RNA dataset	
	Kidney (pTPM)	Glomerulus	Tubules	P- value	Cell type
VEGFA	96.1	medium	medium	0.962	PODO
PTPRO	22.6	high	not detected	0.000339427082146	PODO
LUM	36.3	not detected	not detected	Not detected	Not detected
COL6A3	6.8	not detected	not detected	2.55212579805471E-06	MES
FN1	38.9	medium	high	0.406463264506511	MES
POSTN	23.0	not detected	low	0.010690303094222	MES
KNG1	126.5	medium	high	1.10077051593139E-05	DCT-CT
FGF1	40.9	high	not detected	0.002353864219147	PODO
BMP2	3.3	No data	No data	No data	No data
LOX	18.9	No data	No data	No data	No data
EGF	13.2	No data	No data	0.027696802696135	DCT-CT
DCN	301.3	low	not detected	0.800847912989593	PODO
SPARC	158.7	High	low	0.018698295677998	PODO
VCAN	9.6	not detected	not detected	9.57643667243769E-07	PEC
PLCE1	8.2	Medium	Medium	0.455738614120668	PODO
CTGF	167.1	low	low	0.862180638654418	PEC

Mean TPM of DCN was higher in kidneys with an expression rate of 301.3, followed by SPARC with a value of 158.7. Their expression rates were the highest in the urinary bladder with 1509.2 pTPM and 345.8 pTPM for DCN and SPARC respectively. DCN was identified as a renal cancer prognostic marker with lower levels of its protein identified in glomeruli cells of normal kidneys whereas higher levels of SPARC was predicted in glomeruli and lower levels in tubuli cells of normal kidneys. Higher protein expressions of PTPRO and FGF1 were detected in glomerulus and not in tubuli. PTPRO and FGF1 were already established prognostic biomarkers of renal cancers. Higher protein expression levels of FN1 and KNG1 were identified in normal tubular kidneys and medium levels in glomerulus and bladder tissues. Expressions of LUM, VCAN, COL6A3 were not detected in the normal glomerular, tubular and bladder tissues.

From the overall analysis of hub genes and expression analysis, overexpression of FN1, LUM, VCAN could be the potential prognostic biomarkers of GDKD. Downregulation of KNG1 and upregulation of POSTN and SPARC could be significant biomarkers of TDKD. Upregulated CTGF represents a potential biomarker of EDN. The below cutoff expression (represented in transcripts per kilobase million or TPM) of PTPRO could represent any of the three diseased conditions such as early diabetic nephropathy, glomerular and tubular diabetic kidney diseases. Although the DCN is normally expressed in kidney tissue, high traces of its expression was also found in other organs such as heart and muscles. Thus, despite its significant expression in upregulated DEGs of all three datasets, it cannot be proposed as a suitable biomarker for DKD.

### Comparison of Hub Genes With snRNA Transcriptome Dataset

**Figure 6:**
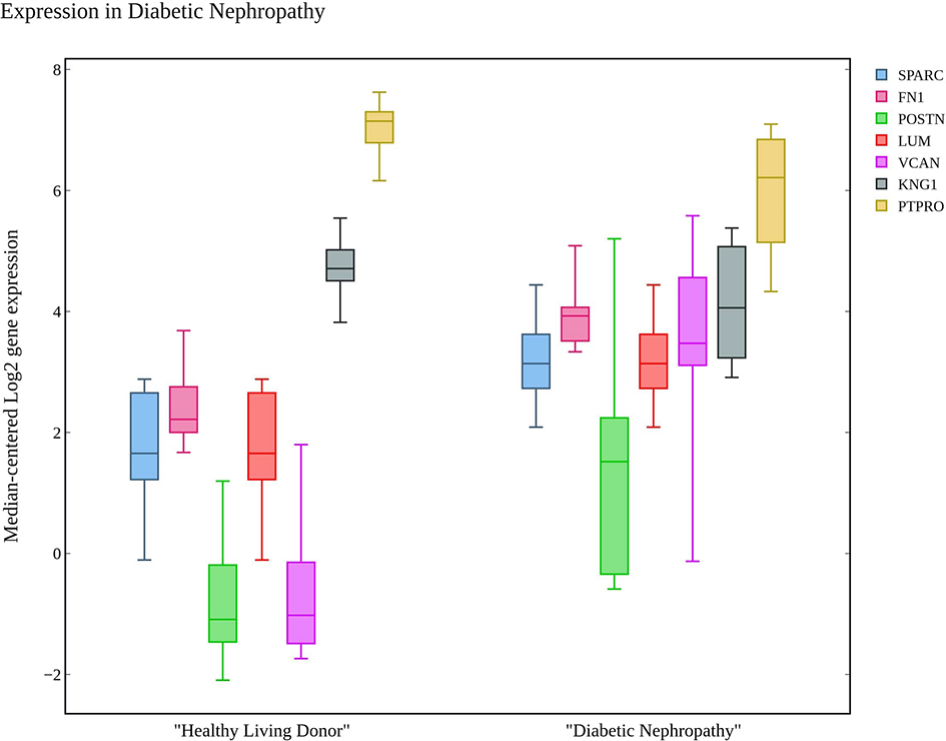
The box plot of seven candidate genes that exhibit the over and under expression rates in diabetic nephropathy in comparison with healthy living donors.

The hub genes were compared with the snRNA transcriptome dataset to infer their potentiality. The genes SPARC, THBS1, EGF, PTPRO, FGF1, POSTN, and COL6A3 were identified to be significantly enriched in the PODO, MES, DCT, PODO, MES, PEC, and MES samples respectively with significant p-value (38) as seen in **Table 2**. LUM, BMP2 and LOX were not detected. VEGFA, FN1, KNG1, FGF1, DCN, VCAN and CTGF were identified in PODO, MES, DCT-CT, PODO, PODO, PEC and PEC respectively. As seen in the **Figure 6**, Nephroseq v5 webserver predicted the over expression of FN1, SPARC, LUM, VCAN, and POSTN with fold change of 2.808, 1.655, 6.25, 1.77, and 3.324 in diabetic nephropathy respectively. Under expression of PTPRO and KNG1 with fold change of -3.308 and -1.618 respectively were reported in diabetic nephropathy in comparison with healthy living donors. These results correlated with the identified putative biomarkers of GDKD, TDKD and EDN except that of CTGF, where it has an under expression (fold change of -4.728) in diabetic nephropathy in contrast to the earlier predictions.

### Drug Perturbation of the Transcriptome Data

Further, we identified the small-molecules chemical perturbagens. The large publicly available gene expression datasets from LINCS were explored to first identify the signature genesets for kidney disease. Subsequently, we identified the 50 different drug perturbation signatures for the overlapping DEGs. We filtered out the similar and opposite signatures for the DEGs from the human renal epithelial cell line HA1E (41). Drugs with similar gene signatures represent the ones strikingly mimic the current gene expression patterns whereas the opposite gene signature patterns represent the ones capable of reversing the observed current expression profiles (42). In this study, the top drugs were ranked based on the similarity scores. Two drugs viz. pidorubicine, a topoisomerase inhibitor (LINCS ID- BRD-K04548931) and Polo-like kinase inhibitor (LINCS ID- BRD-K41652870) were identified from the opposite signatures. Both the drugs were validated to be effective on the renal epithelium cell line with the similarity score of -0.9812 and -0.9516, respectively. The details of the drug perturbation analysis is presented in **Table 3**. The transcriptomic signatures and the action of the drugs BRD-K04548931 and BRD-K41652870 on renal epithelium cell lines (HA1E) are shown in **Supplementary Figure 3**.

**Table 3:** Drug perturbation analysis of the overlapped DEGs.

Signature ID	Pubchem ID	Drug	Similarity score	Drug Structure
Similar signatures - drugs mimic the originally observed expression patterns				
CPC002_HA1E_6H:BRD-K80348542-001-01-4:10	442195 Cephaeline	BRD-K80348542	1	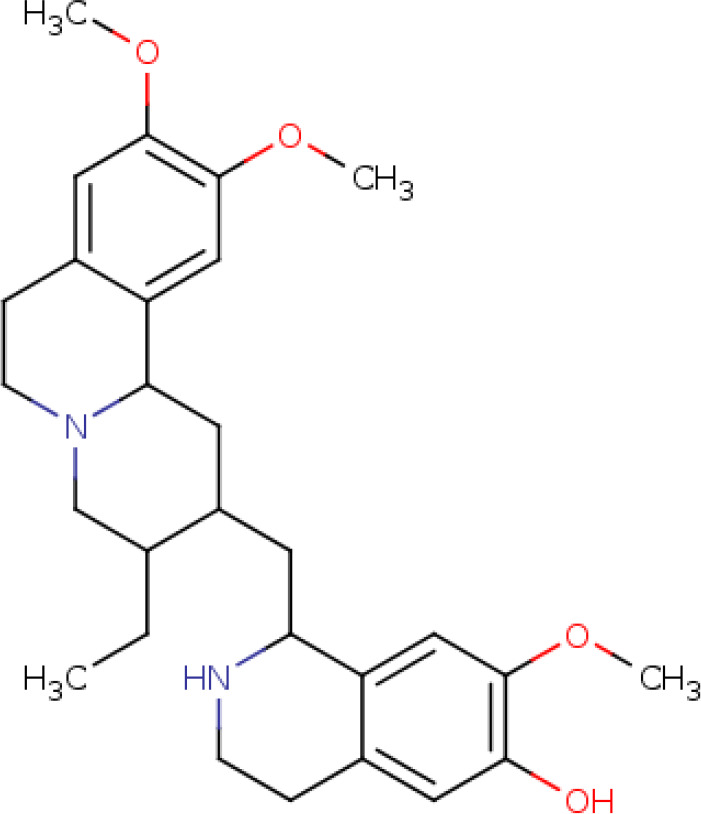
CPC010_HA1E_6H:BRD-A24643465-001-05-3:10	16219462 Homoharringtonine	BRD-A24643465 protein synthesis inhibitor	1	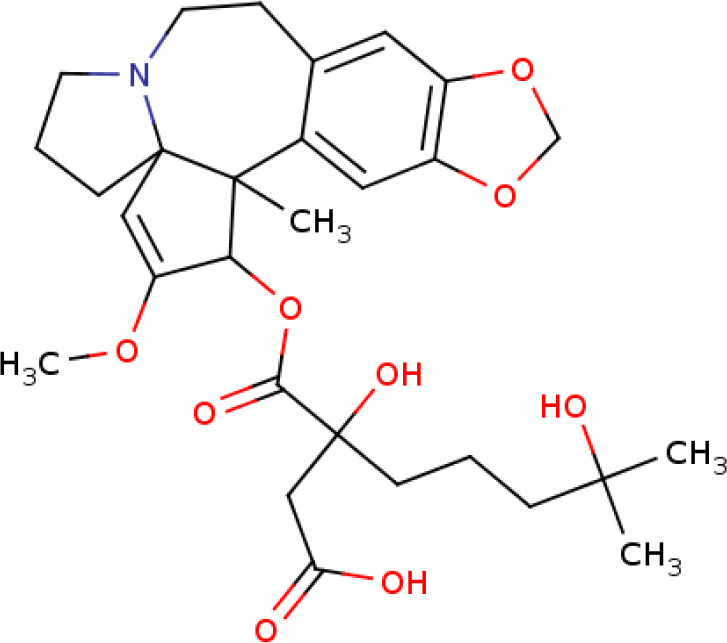
Opposite signatures - drugs reverse the observed pattern of DEGs				
CPC002_HA1E_24H:BRD-K91370081-001-10-3:10	65348 Pidorubicine	BRD-K04548931 Topoisomerase inhibitor	-0.9812	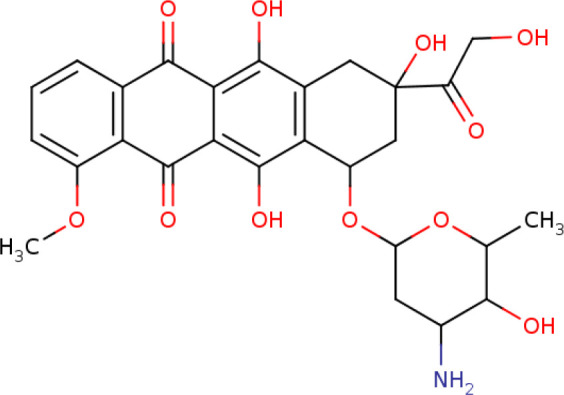
CPC012_HA1E_6H:BRD-K41652870-001-01-9:10	3190037	BRD-K41652870 Polo-like kinase inhibitor	-0.9516	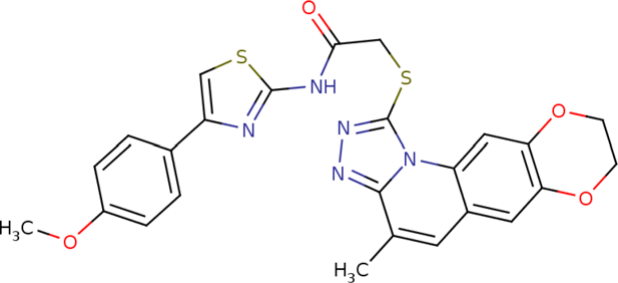

## Discussion

Currently, due to the aggressive comorbidities associated with the DKD poses an enormous challenge in their diagnosis. The identification of the better biomarkers are urgently needed to improve diagnosis. Niewczas et al. recently examined the proteomic profiling of circulating proteins (19) in type 1 and type 2 diabetes patients. They identified an extremely robust inflammatory signature, consisting of 17 proteins enriched for TNF Receptor Superfamily members with a 10-year risk of end-stage renal disease. Mitrofanova et al., reported that SMPDL3b modulates insulin receptor signaling in DKD (43). Bjornstad et al., recently reported the relationships between markers of tubular injury and intrarenal hemodynamic function in adults with and without type 1 diabetes (44). Hirao et al., reported the discovery of a useful biomarker for EDN discovery by proteomics analysis of urine proteomes of DM patients without microalbuminuria and healthy volunteers (45). Cheung et al. performed an exome-chip association analysis in Chinese subjects with type 2 diabetes (46). They noticed elevated circulating pigment epithelium-derived factor levels were associated with increased risks of DN and sight-threatening diabetic retinopathy. Collectively, the identification of novel markers for prognosis and diagnosis of DKD is an active area of research.

In this work, we aimed to reanalyze the omics molecular profiles available in the public databases to identify the prognosis biomarkers and small-molecules chemical perturbagens of the DKD. Similar approaches have been advocated in previous studies. In the current study, we integrated gene expression patterns of three stages of DKD viz. EDN, GDKD and TDKD. We obtained three datasets of gene expression patterns from NCBI-GEO: GSE111154 with samples affected by EDN along with healthy control, GSE30528 with samples from the affected GDKD along with healthy control, and finally GSE30529 containing samples affected with TDKD along with healthy control.

A total of 280 up- and 27 downregulated DEGs were identified and the subsequent analysis was carried out on them. Gene annotations of DEGs revealed the upregulations of biological processes such as cell migration, cytokine stimulation, and glomerular epithelial cell differentiation. Downregulated molecular functions being metallopeptidase inhibitor, VEGFA receptor binding and integrin binding ability. Cellular components like phagocytic vesicles and granular lumen were found in higher levels. Pathways enrichment analysis disclosed potential activation and aggregation of platelets, collagen fiber formation, hemostasis and activated complement cascade. Pathways involving the vascular endothelial growth factor dimerization and its interaction with ligands were substantially deferred from the normal occurrences. The subsequent inter-relational analysis supported the above findings. Strikingly, reduced developments of glomeruli and nephrons were noticed in all three diseased conditions. The determination of PPI networks allowed us to narrow down the DEGs to identify potential biomarkers.

We compared the gene expression levels of the identified hub genes. FN1 (responsible for cell adhesion), LUM and VCAN (veriscan) (involved in cytokine activity and signaling pathways) could be the putative upregulated biomarkers of GDKD. We predicted the lower expression of KNG1 (kininogen1) responsible for elevated cytosolic calcium levels and fibrin clots can be a putative prognostic biomarker for TDKD. Upregulation of POSTN (peristin) and SPARC (secreted protein acidic and cysteine rich) could also be putative biomarkers of TDKD. PTPRO (protein tyrosine phosphatase receptor O) responsible for the chemotaxis and regulation of glomerular filtration was proposed as an attractive downregulated candidate biomarker for all three diseases. The genes FN1, and PTPRO were reported by the Online Mendelian Inheritance in Man (OMIM) database to be associated with glomerulopathy, renal fibrosis and nephrotic syndrome, respectively (47). Correlation of expression of the candidate genes were obtained in comparison to the snRNA datasets, except that of CTGF which had a contradictory under expression in diabetic nephropathy Thus, the identified key candidate genes - FN1, SPARC, VCAN, LUM, POSTN, KNG1 and PTPRO can be considered further for the investigation in clinical research. Finally the two drugs viz. pidorubicine, a topoisomerase inhibitor (LINCS ID- BRD-K04548931) and Polo-like kinase inhibitor (LINCS ID- BRD-K41652870) were also identified using the induced transcriptional gene signature database. These drugs were known to have the validated role in reversing the differential gene expression patterns observed in all three datasets (48).

Though the study has integrated gene enrichment, pathways, tissue and cell specific expression analysis in the identification of hub genes for diabetic kidney conditions, there exists few limitations as well. There are advanced sc-RNA and bulk sequencing technologies available, yet the current study relied upon the affymetrix microarray datasets to infer the diabetic kidney disease pathogenesis for three reasons: first, majority of the diabetic kidney disease datasets were based on affymetrix platform, secondly, the human transcriptome data were limited and finally, the datasets without any treatment conditions were a little. The present study needs extensive clinical validation of the proposed candidate genes and drug perturbations. Thus the field demands more research and the current research opens up novel directions to explore the biomarkers and pathology of diabetic kidney diseases. Comparison of the hub genes with the sn-RNA sequence data added confirmation and correlated with the main findings of the study.

In conclusion, we have investigated the potential biomarkers/key candidate genes for DKD using the integrated bioinformatics analysis. We identified seven candidate genes FN1, LUM, VCAN, KNG1, POSTN, SPARC and PTPRO significantly associated with the progression of DKD, and further investigation of these genes in clinical research is warranted. Furthermore, the results of this study increase our understanding of the molecular drivers, critical genes and pathways that underlie DKD initiation and progression. Identification of the small-molecules chemical perturbagens further validate their potentials as therapeutic targets.

## Data Availability Statement

The original contributions presented in the study are included in the article/**Supplementary Material**. Further inquiries can be directed to the corresponding author.

## Author Contributions

ZG designed the study, curated the datasets, analyzed the computational results and wrote the original draft. AS helped in the acquisition, analysis and interpretation of the datasets, and wrote the revised manuscript. XML, XLL and LS helped in the datasets analysis and wrote the original manuscript. All authors contributed to the article and approved the submitted version.

## Funding

This work is funded by the Everest Project of Air Force Medical University (NO: 2020ZFB002).

## Conflict of Interest

The authors declare that the research was conducted in the absence of any commercial or financial relationships that could be construed as a potential conflict of interest.

## Publisher’s Note

All claims expressed in this article are solely those of the authors and do not necessarily represent those of their affiliated organizations, or those of the publisher, the editors and the reviewers. Any product that may be evaluated in this article, or claim that may be made by its manufacturer, is not guaranteed or endorsed by the publisher.

## Supplementary Material

Supplementary Figure 1 Gene Ontology (GO) term enrichment analysis of upregulated genes in the three datasets (EDN: GSE111154, GDKD: GSE30528 and TDKD: GSE30529). GO analysis of the upregulated genes were examined for three sub-ontologies (biological process, molecular function, and cellular component).

Supplementary Figure 2 Gene Ontology (GO) term enrichment analysis of downregulated genes in the three datasets (EDN: GSE111154, GDKD: GSE30528 and TDKD: GSE30529). GO analysis of the downregulated genes were examined for three sub-ontologies (biological process, molecular function, and cellular component).

Supplementary Figure 3 Signaling pathway enrichment analysis of overlapped DEGs. from KEGG and REACTOME pathways.
