# Supplementary figures and images for: Identification of Key Candidate Genes and Chemical Perturbagens in Diabetic Kidney Disease Using Integrated Bioinformatics Analysis
Source: Front Endocrinol (Lausanne). 2021 Sep 7;12:721202. doi: 10.3389/fendo.2021.721202 (PMC8453249; doi:10.3389/fendo.2021.721202)

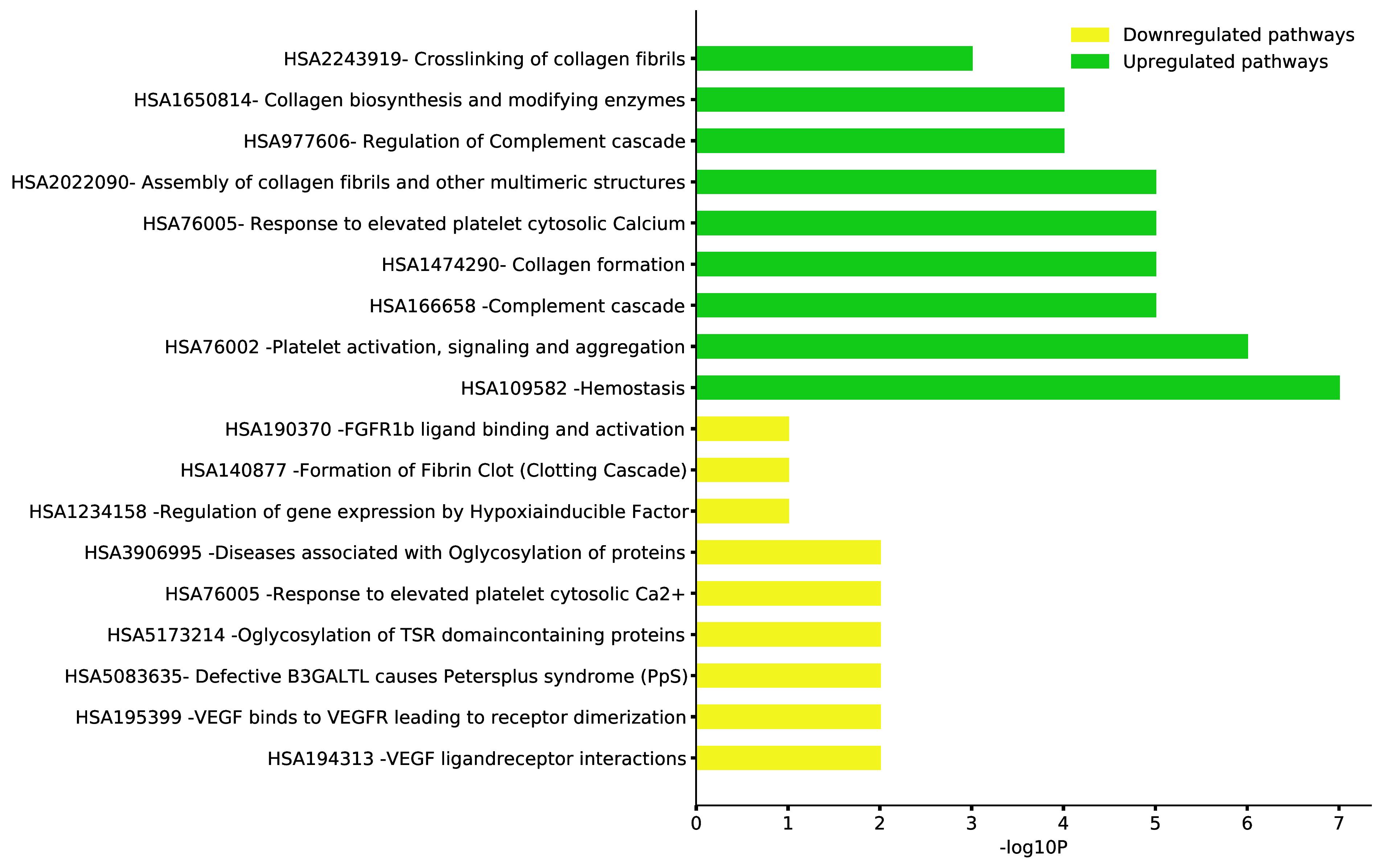

Supplement: Supplementary Figure 1 — Gene Ontology (GO) term enrichment analysis of upregulated genes in the three datasets (EDN: GSE111154, GDKD: GSE30528 and TDKD: GSE30529). GO analysis of the upregulated genes were examined for three sub-ontologies (biological process, molecular function, and cellular component). [file Image_1.jpeg]

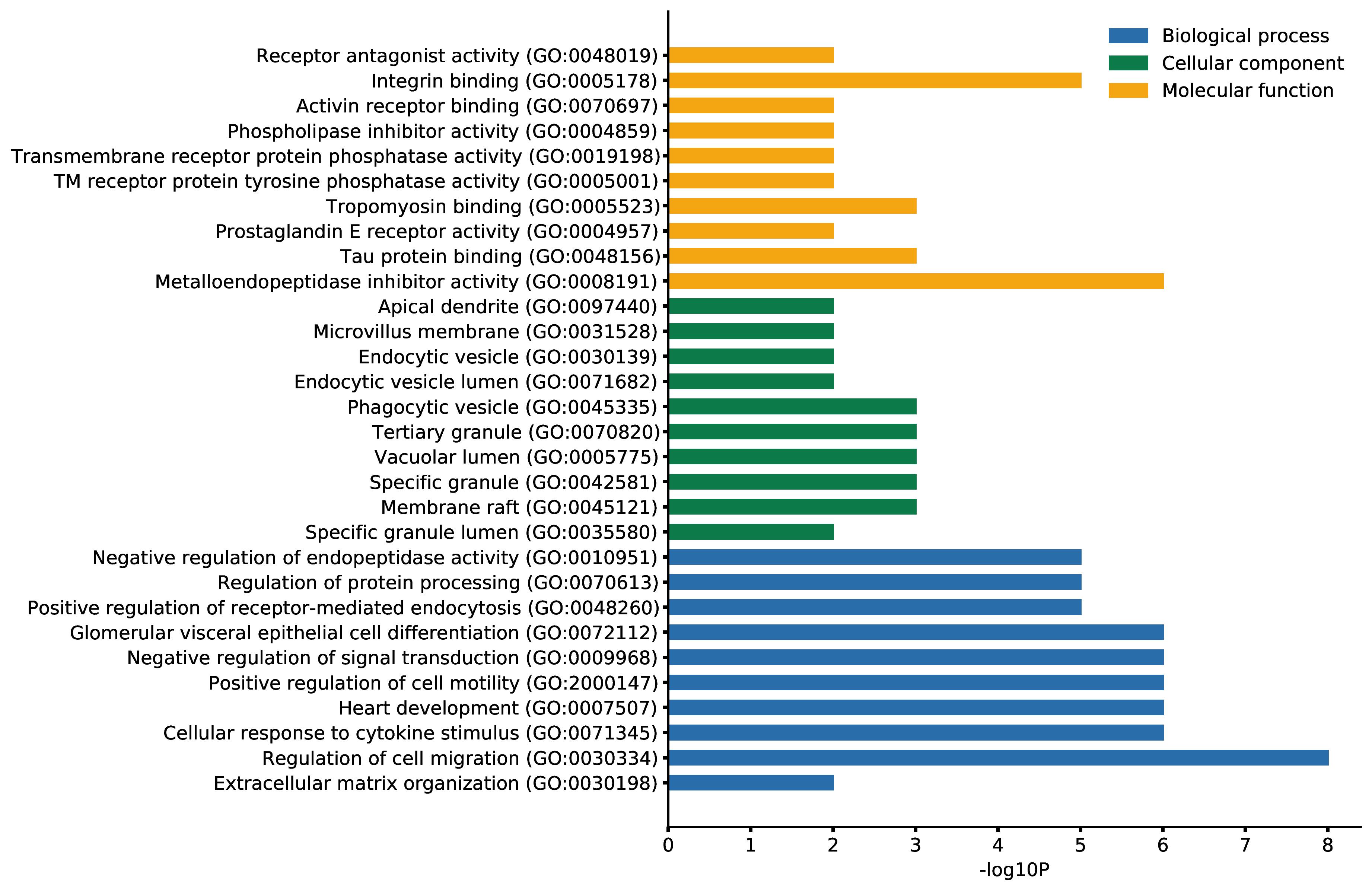

Supplement: Supplementary Figure 2 — Gene Ontology (GO) term enrichment analysis of downregulated genes in the three datasets (EDN: GSE111154, GDKD: GSE30528 and TDKD: GSE30529). GO analysis of the downregulated genes were examined for three sub-ontologies (biological process, molecular function, and cellular component). [file Image_2.jpeg]

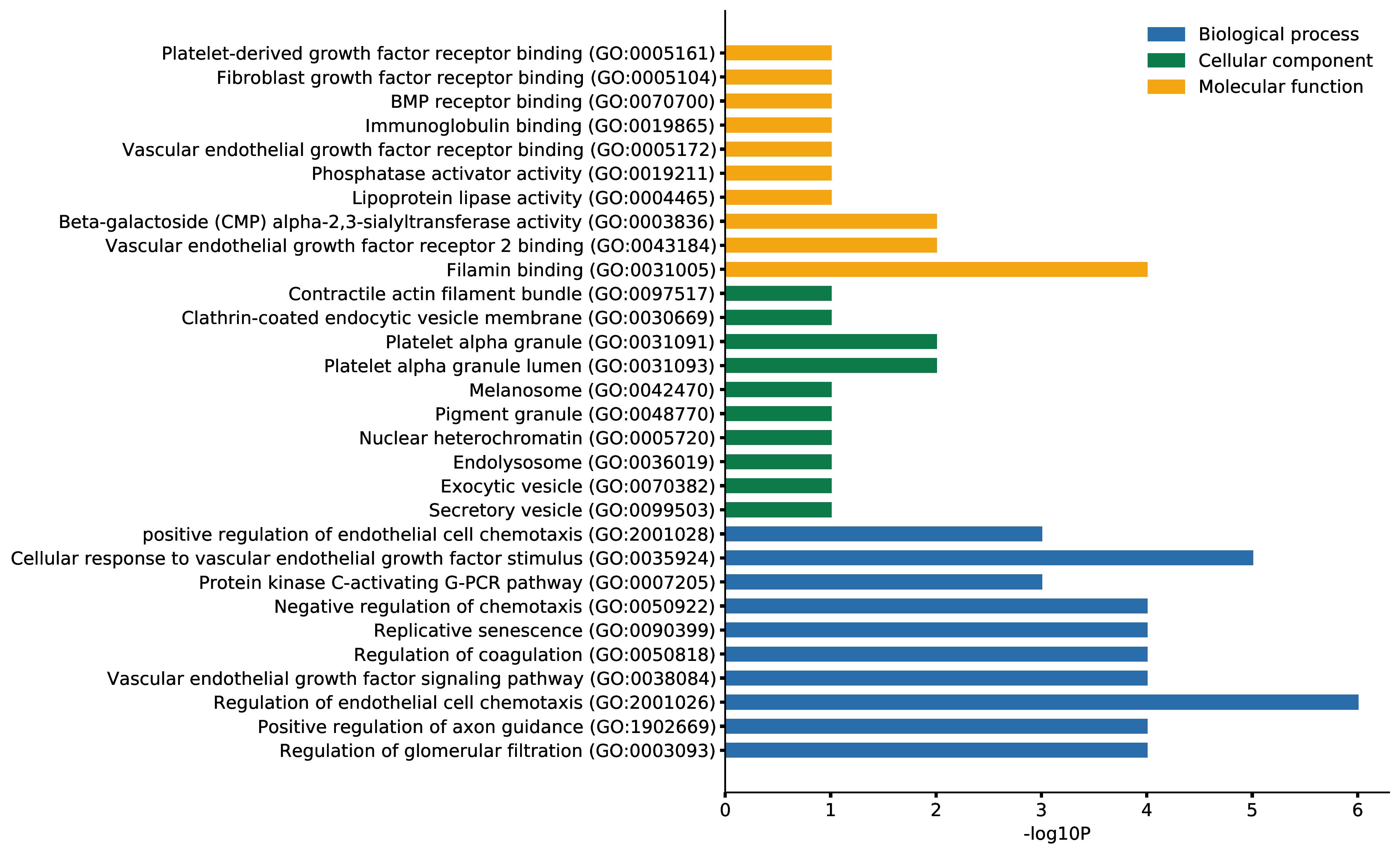

Supplement: Supplementary Figure 3 — Signaling pathway enrichment analysis of overlapped DEGs. from KEGG and REACTOME pathways. [file Image_3.jpeg]
