# Supplementary material for: Identification of Key Candidate Genes and Chemical Perturbagens in Diabetic Kidney Disease Using Integrated Bioinformatics Analysis
Source: Front Endocrinol (Lausanne). 2021 Sep 7;12:721202. doi: 10.3389/fendo.2021.721202 (PMC8453249; doi:10.3389/fendo.2021.721202)
Supplement: Supplementary file 4 [file Table_1.docx]

**Supplementary Table 1.** Gene ontology term enrichments of the overlapping DEGs.

| **Term** | **Description** | **Count** | **P- value** |
| --- | --- | --- | --- |
| **Upregulated gene ontology terms** | | | |
| GO:0030198 | Extracellular matrix organisation | 2 | 1.65 x 10^-13^ |
| GO:0030334 | Regulation of Cell migration | 8 | 7.538 x 10^-10^ |
| GO:0071345 | Cellular response to cytokine stimulus | 6 | 1.31 x 10^-7^ |
| GO:0007507 | Heart development | 6 | 1.843 x 10^-7^ |
| GO:2000147 | Positive regulation of cell motility | 6 | 2.413 x 10^-7^ |
| GO:0009968 | Negative regulation of signal transduction | 6 | 5.53 x 10^-7^ |
| GO:0072112 | Glomerular visceral epithelial cell differentiation | 6 | 6.092 x 10^-7^ |
| GO:0048260 | Positive regulation of receptor mediated endocytosis | 5 | 1.463 x 10^-6^ |
| GO:0070613 | Regulation of protein processing | 5 | 1.903 x 10^-6^ |
| GO:0010951 | Negative regulation of endopeptidase activity | 5 | 2.416 x 10^-6^ |
| GO:0035580 | Specific granule lumen | 2 | 2.273 x 10^-4^ |
| GO:0045121 | Membrane raft | 3 | 2.739 x 10^-4^ |
| GO:0042581 | Specific granule | 3 | 4.345 x 10^-4^ |
| GO:0005775 | Vacuolar lumen | 3 | 4.547 x 10^-4^ |
| GO:0070820 | Tertiary granule | 3 | 5.2 x 10^-4^ |
| GO:0045335 | Phagocytic vesicle | 3 | 1.093 x 10^-3^ |
| GO:0071682 | Endocytic vesicle lumen | 2 | 1.896 x 10^-3^ |
| GO:0030139 | Endocytic vesicle | 2 | 4.15 x 10^-3^ |
| GO:0031528 | Microvillus membrane | 2 | 5.172 x 10^-3^ |
| GO:0097440 | Apical dendrite | 2 | 5.172 x 10^-3^ |
| GO:0008191 | Metalloendopeptidase inhibitor activity | 6 | 3.793 x 10^-7^ |
| GO:0048156 | Tau protein binding | 3 | 1.443 x 10^-4^ |
| GO:0004957 | Prostaglandin E receptor activity | 2 | 2.823 x 10^-3^ |
| GO:0005523 | Tropomyosin binding | 3 | 8.814 x 10^-3^ |
| GO:0005001 | TM receptor protein tyrosine phosphatase activity | 2 | 1.09 x 10^-3^ |
| GO:0019198 | Transmembrane receptor protein phosphatase activity | 2 | 1.09 x 10^-3^ |
| GO:0004859 | Phospholipase inhibitor activity | 2 | 5.172 x 10^-3^ |
| GO:0070697 | Activin receptor binding | 2 | 5.172 x 10^-3^ |
| GO:0005178 | Integrin binding | 5 | 6.836 x 10^-6^ |
| GO:0048019 | Receptor antagonist activity | 2 | 1.596 x 10^-3^ |
| **Downregulated gene ontology terms** | | | |
| GO:0003093 | Regulation of glomerular filtration | 4 | 2.43 x 10^-5^ |
| GO:1902669 | Positive regulation of axon guidance | 4 | 2.43 x 10^-5^ |
| GO:2001026 | Regulation of endothelial cell chemotaxis | 6 | 5.529 x 10^-7^ |
| GO:0038084 | Vascular endothelial growth factor signaling pathway | 4 | 3.399 x 10^-5^ |
| GO:0050818 | Regulation of coagulation | 4 | 7.266 x 10^-5^ |
| GO:0090399 | Replicative senescence | 4 | 8.873 x 10^-5^ |
| GO:0050922 | Negative regulation of chemotaxis | 4 | 8.873 x 10^-5^ |
| GO:0007205 | Protein kinase C-activating G-PCR pathway | 3 | 1.333 x 10^-7^ |
| GO:0035924 | Cellular response to vascular endothelial growth factor stimulus | 5 | 3.072 x 10^-7^ |
| GO:2001028 | positive regulation of endothelial cell chemotaxis | 3 | 1.333 x 10^-7^ |
| GO:0099503 | Secretory vesicle | 1 | 2.061 x 10^-2^ |
| GO:0070382 | Exocytic vesicle | 1 | 2.061 x 10^-2^ |
| GO:0036019 | Endolysosome | 1 | 2.569 x 10^-2^ |
| GO:0005720 | Nuclear heterochromatin | 1 | 2.569 x 10^-2^ |
| GO:0048770 | Pigment granule | 1 | 2.696 x 10^-2^ |
| GO:0042470 | Melanosome | 1 | 2.696 x 10^-2^ |
| GO:0031093 | Platelet alpha granule lumen | 2 | 7.776 x 10^-3^ |
| GO:0031091 | Platelet alpha granule | 2 | 6.067 x 10^-3^ |
| GO:0030669 | Clathrin-coated endocytic vesicle membrane | 1 | 5.075 x 10^-2^ |
| GO:0097517 | Contractile actin filament bundle | 1 | 5.075 x 10^-2^ |
| GO:0031005 | Filamin binding | 4 | 7.266 x 10^-5^ |
| GO:0043184 | Vascular endothelial growth factor receptor 2 binding | 2 | 7.776 x 10^-3^ |
| GO:0003836 | Beta-galactoside CMP alpha-2,3-sialyltransferase activity | 2 | 9.066 x 10^-3^ |
| GO:0004465 | Lipoprotein lipase activity | 1 | 1.035 x 10^-2^ |
| GO:0019211 | Phosphatase activator activity | 1 | 1.164 x 10^-2^ |
| GO:0005172 | Vascular endothelial growth factor receptor binding | 1 | 1.164 x 10^-2^ |
| GO:0019865 | Immunoglobulin binding | 1 | 1.421 x 10^-2^ |
| GO:0070700 | BMP receptor binding | 1 | 5.075 x 10^-2^ |
| GO:0005104 | Fibroblast growth factor receptor binding | 1 | 1.549 x 10^-2^ |
| GO:0005161 | Platelet-derived growth factor receptor binding | 1 | 5.075 x 10^-2^ |
